# Pathogenic BALB/c mice infection model for evaluation of mpox countermeasures

**DOI:** 10.1038/s41421-024-00739-z

**Published:** 2024-10-13

**Authors:** Lin Cheng, Wenqi Huang, Meimei Duan, Zhuohuan Li, Qi Chen, Mingxia Zhang, Zheng Zhang

**Affiliations:** 1 Institute for Hepatology, National Clinical Research Center for Infectious Disease, Shenzhen Third People’s Hospital; the Second Affiliated Hospital, School of Medicine, Southern University of Science and Technology, Shenzhen, Guangdong China; 2 School of Medicine, The Chinese University of Hong Kong, Shenzhen, Guangdong China

**Keywords:** Innate immunity, Immunology

The 2022 outbreak of the mpox (also known as monkeypox) rapidly spread globally, resulting in 106, 310 confirmed cases as of Aug 2024, spanning 123 countries. Although the public health emergency of international concern was discontinued in May 2023, mpox suddenly broke out one month later and spread rapidly across the mainland of China, resulting in over 1000 confirmed cases within three months. More worrying, a large-scale independent outbreak, caused by a more virulent clade I mpox virus (MPXV), emerged in the Democratic Republic of Congo in 2023. These outbreaks highlight the critical need to develop additional mpox countermeasures.

Animal models are crucial for evaluating the efficacy of MPXV vaccines and antiviral therapeutic agents. Nonhuman primates have pathogenesis and symptoms features most similar to those of humans when infected with MPXV, but are too expensive to be extensively used. Rodent models are cheaper, easier and less dangerous to handle. Therefore, the highly susceptible CAST/EiJ mice model has been widely used in developing mpox countermeasures^1–3^. However, the supply of CAST/EiJ mice primarily bred in Jackson laboratory is limited. Meanwhile, vaccinia virus (VACV), another Orthopoxvirus as MPXV, is capable of triggering lethal infections in BALB/c mice and a biosafety level-3 laboratory is not required. For these reasons, effective drugs, neutralizing antibodies, and vaccines against mpox developed after the 2022 outbreak were mostly evaluated using the vaccinia virus infection of the BALB/c mice model^2,4–6^.

We have previously isolated and characterized an MPXV clade IIb strain SZTH42 from a 37-year-old male mpox patient in 2023^7^. We attempted to infect BALB/c mice intranasally with the MPXV/SZTH42 and then monitored daily for symptoms. Surprisingly, mice in the infected group exhibited ruffled fur from 3 days post-infection (dpi), followed by a hunched posture. Unexpectedly, mice lost ~30% of their initial body weight at 7 dpi (Supplementary Fig. S1). This result contradicts the widely accepted concept that BALB/c mice are not susceptible to MPXV. Earlier research showed that intranasal infection with a more virulent MPXV clade I isolate led to weight loss and death in CAST/EiJ mice. In contrast, there were no deaths of BALB/c mice at a 10, 000-fold higher dose, with the maximum weight loss of only 20%^1^. More recently, BALB/c mice inoculated intranasally with over 4 × 10^6^ plaque forming unit (PFU) of a less virulent MPXV clade IIb isolate showed no clinical symptoms and weight loss^8^.

To confirm the pathogenic phenotype of BALB/c mice, we repeated the experiment with doses of MPXV/SZTH42 ranging from 0.5 × 10^5^ to 4 × 10^5^ focus forming unit (FFU). Consistently, mice infected with 4 × 10^5^ FFU resulted in rapid and uniform weight loss with ignorable error bars (Fig. 1a). This group of mice was all euthanized at 6 or 7 dpi while the weight loss exceeded 30% of their initial weight (Fig. 1b). One mouse that received 2 × 10^5^ FFU showed rapid weight loss and was euthanized at 6 dpi, whereas the other four mice exhibited maximum weight loss of 17%, and they rebounded at 4 dpi and recovered the lost weight (Supplementary Fig. S2). All animals infected with 1 × 10^5^ FFU or less showed no signs of disease. Furthermore, MPXV proliferated rapidly in mice infected with 4 × 10^5^ FFU, and the infectious virions in the lungs increased by about 250 times at 7 dpi compared with that at 1 dpi (Fig. 1c). No viral DNA and infectious virion were detected in other organs including the liver, spleen, kidneys, and ovaries (data not shown). Notably, the percentage of weight loss in mice infected with 3 × 10^5^ FFU under mild anesthesia for 4 days is significantly positively correlated with lung viral loads (Fig. 1d). Besides, the MPXV-specific antibody levels and serum neutralizing activities in mice rapidly increased and peaked at 21 dpi (Fig. 1e, f). These results demonstrate that the observed pathogenic phenotypes of BALB/c mice are indeed caused by MPXV infection.

**Fig. 1:**
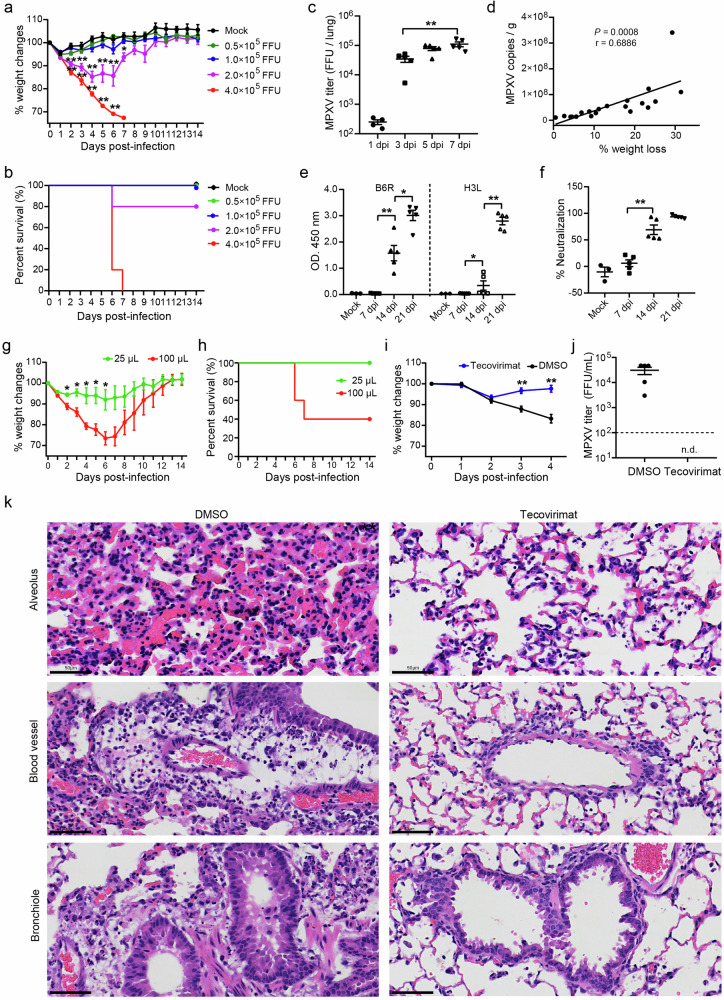
Pathogenic infection model of MPXV/SZTH42 in BALB/c mice for antiviral drugs evaluation. **a**–**c** Groups of 6- to 8-week-old female BALB/c mice were infected intranasally with doses of MPXV/SZTH42 between 0.5 × 10^5^ and 4 × 10^5^ FFU in total volume of 50 μL (*n* = 5). Control mice were mock infected with virus diluent (*n* = 3). Animals were monitored for weight loss (**a**) and death (**b**) for 14 days post-infection. Infectious virions in the lungs of mice infected with 4 × 10^5^ FFU were titrated at indicated time points (**c**). **d** Mice were intranasally infected with 3 × 10^5^ FFU under mild anesthesia (*n* = 20). The correlation between the percentage of weight loss and lung viral loads determined by qPCR was analyzed at 4 dpi. **e**, **f** Groups of mice were intranasally infected with 3 × 10^5^ FFU (*n* = 5) or virus dilute (Mock, *n* = 3). Levels of B6R- and H3L-specific antibodies (**e**) and serum neutralizing activity (**f**) were determined by ELISA and Focus Reduction Neutralization Test (FRNT), respectively. **g**, **h** Groups of mice were intranasally infected with 2 × 10^5^ FFU in total volume of 25 μL or 100 μL (*n* = 5). Animals were monitored for weight loss (**g**) and death (**h**) for 14 days post-infection. **i**–**k** Mice were infected with 4 × 10^5^ FFU and treated via oral gavage from the challenge day with tecovirimat (10 mg/kg) or DMSO daily for 4 consecutive days (*n* = 5). Weight changes were monitored for 4 days (**i**), and infectious MPXV titers in lungs at 4 dpi were determined by focus-forming assay (**j**). The dashed line represents the limit of detection of the assay. n.d., not detected. Representative images of hematoxylin-eosin (H&E) staining of lung tissue sections at 4 dpi (**k**). Scale bars, 50 μm. Error bars indicate SEM (**a**, **c**, **e**–**g**, **i**, **j**). Statistical significance was evaluated using Student’s *t*-test (**a**, **c**, **e**–**g**, **i**). *P < 0.05, **P < 0.01.

Previous studies have shown that BALB/c mice intranasally infected with MPXV clade IIb isolate showed no signs of disease and weight loss^8^. However, our data demonstrated that MPXV clade IIb isolate is capable of causing pathogenic infections in BALB/c mice. To exclude the possibility of MPXV strain-specific pathogenic infection in BALB/c mice, two additional MPXV clade IIb isolates SZTH41 and SZTH45 from different mpox patients were applied for intranasal infection of BALB/c mice. Compared with MPXV/SZTH42, mice infected with SZTH41 and SZTH45 exhibited similar symptoms and comparable weight loss (Supplementary Figs. S3, S4), indicating a universal pathogenicity of MPXV IIb stains in BALB/c mice, which is contrary to previous understanding.

The volume of the inoculum and the level of anesthesia might be key factors contributing to this discrepancy. The volume of intranasal inoculum was 50 μL in this study but ranged from 10 μL to 20 μL in previous studies^1,8,9^. The delivery volume and anesthesia depth significantly affect the distribution of inoculum in the respiratory tract, and a larger volume plus deep anesthesia contributes to direct delivery into the lower respiratory tract of mice^10^. To further verify the effect of inoculum volume on the pathogenicity, groups of mice under deep anesthesia were intranasally infected with 2 × 10^5^ FFU MPXV/SZTH42 in volume of either 25 μL or 100 μL, respectively. After the 25 μL challenge all animals survived, and the average weight loss did not exceed 8% of the starting weight. In striking contrast, mice received the same dose but in volume of 100 μL resulted in rapid weight loss, and three mice were euthanized at 6 or 7 dpi due to weight loss exceeding 30% (Fig. 1g, h). In addition, the maximum weight loss of the remaining two mice was 25% and 18%, respectively (Supplementary Figs. S5, S6). These results highlight the impact of inoculum volume on the pathogenesis caused by the intranasal route of infection, further confirming our finding that MPXV clade IIb isolate is capable of causing pathogenic infections in BALB/c mice.

The volumes delivered intranasally in mice varied greatly, ranging from a total of 2 μL to 150 μL^11^ given as a single bolus. To efficiently deliver the inoculum into mouse lungs, the intranasal instillation required a dose volume of 50 µL or more^12^. But no further increase in the relative distribution to the lungs for inoculum occurred with the instillation of 75 μL compared with 50 µL^10^. Taking into account both ethical and scientific considerations, mice under appropriate anesthesia were intranasally challenged with 50 μL of MXPV for future experiments.

To assess the BALB/c-MPXV model for evaluation of therapeutics, we determined the ability of tecovirimat (also known as TPOXX and ST-246), an inhibitor of Orthopoxvirus p37 which is involved in the release of enveloped virus from the cell and subsequent dissemination, to protect against a pathogenic challenge with MPXV. Groups of BALB/c mice were intranasally instilled with 4 × 10^5^ FFU MPXV/SZTH42 in a volume of 50 μL per animal. Mice were treated for four consecutive days beginning from the day of inoculation (day 0) with tecovirimat (10 mg/kg) or DMSO. Control mice lost weight rapidly and continuously from 1 dpi, whereas the body weight of tecovirimat-treated mice rebounded from 2 dpi and recovered at 4 dpi (Fig. 1i). Subsequently, animals were euthanized at 4 dpi to examine pulmonary MPXV titers and histopathological changes. Control mice had mean MPXV titers of over 3 × 10^4^ FFU/mL, whereas infectious MPXV in the lungs of tecovirimat-treated mice was not detected (Fig. 1j). Histopathologically, mice treated with tecovirimat maintained a normal lung structure with mild infiltration of lymphocytes and macrophages into alveolar spaces and around blood vessels. By contrast, control animals showed severe pulmonary interstitial widening with capillary congestion, obvious perivascular edema with extensive lymphocyte infiltration, as well as sharply narrowed and twisted bronchial lumen with a large amount of exudate and bronchiolar epithelial hyperplasia (Fig. 1k; Supplementary Fig. S7). Overall, our data suggest that tecovirimat treatment of MPXV-infected BALB/c mice markedly reduces viral replication in the lungs, leading to rapid viral clearance, obviously improved pulmonary pathological changes, and a rapid rebound of body weight.

In summary, we have reported a pathogenic BALB/c mouse model induced by infection with MPXV/SZTH42, a clade IIb isolate in response to the 2022/2023 outbreak of mpox. After intranasal instillation, BALB/c mice exhibited symptoms such as ruffled fur, hunched back, decreased mobility, as well as over 30% weight loss, and pulmonary histopathological changes, which were cured by tecovirimat treatment. The novel BALB/c-MPXV model would be conducive to filling the gap caused by an insufficient supply of CAST/EiJ mice and pave the way for more extensive research and development of antiviral drugs and vaccines against MPXV in the future.

## Supplementary information

Supplementary Information
