# Supplementary material for: Pathogenic BALB/c mice infection model for evaluation of mpox countermeasures
Source: Cell Discov. 2024 Oct 13;10:105. doi: 10.1038/s41421-024-00739-z (PMC11471750; doi:10.1038/s41421-024-00739-z)
Supplement: Supplementary file 1 — Supplementary Information [file 41421_2024_739_MOESM1_ESM.pdf]

Supplementary Information

Materials and Methods

Cells and antiviral compound. Vero E6 cells were maintained at 37°C and 5% CO_2 in modified Eagle minimal essential medium supplemented with L-glutamine (2 mM), penicillin (100 U/mL), streptomycin (100 μg/mL), HEPES (10 mM) and 10% fetal bovine serum (complement medium), or 2% fetal bovine serum (maintenance medium). Tecovirimat was purchased from Sigma-Aldrich (Cat. SML3863), dissolved at 20 mg/mL in DMSO, and stocked in aliquots at -20 ºC.

Viruses. The MPXV strain hMpxV/China/SZ-SZTH42/2023 (referred to as SZTH42, GISAID accession ID: EPI_ISL_18213374), SZTH41 (GISAID accession ID: EPI_ISL_ 18213375), SZTH45 (GISAID accession ID: EPI_ISL_ 19032701), isolated from male mpox patients aged 37, 42, and 30 in Shenzhen China, respectively, represents the clade IIb that caused the 2022 pandemic. Viruses were grown and titrated in Vero E6 cells. All experiments with live MPXV were performed in a Biosafety Level 3 (BSL-3) facility following standard biosafety practices.

Inoculation of animals. Female BALB/c mice, 6 to 8 weeks of age, were obtained from GemPharmatech Co., Ltd (Guangdong, China). After arrival, all mice were acclimated for 5 to 7 days before the beginning of any experimental procedures and then randomly assigned to experimental groups. On the day of infection, MPXV was diluted in a maintenance medium. Animals were anesthetized by intraperitoneal injection of 1.25% tribromoethanol solution with a dosage of 0.2 mL/10g (Nanjing Aibei Biotechnology). The depth of anesthesia was assessed, including factors such as respiration rate and response to toe pinch (pedal withdrawal reflex), to ensure deep anesthesia prior to infection. Intranasal (i.n.) infections were performed by introduction of 20-100 μL of virus into one nostril. Mock-infected control animals were similarly

inoculated with an equivalent volume of diluent. After infection, animals were monitored daily for any adverse events or clinical signs, and euthanized by the study's endpoint on day 14 after infection. Mice that lost 30% of their starting weight were euthanized in accordance with the Institutional Animal Care and Use Committee.

For treatment experiments, mice were given 10 mg/kg of tecovirimat in 0.2 mL via oral gavage once daily for 4 days beginning on the day of infection (day 0). On day 0, after oral gavage mice were fasted for 6 hours before anesthesia and inoculation. Animals were euthanized on day 4 after infection for the determination of pulmonary viral titer and pathological analysis. Control animals received the same volume of vehicle via the same method of gavage.

Animal ethics statement. Mice were housed in independent ventilation cages and utilized at three to five mice per treatment group. All animals were given food and water ad libitum throughout all experiments. All efforts were made to minimize animal suffering and to reduce the number of animals used. All animal procedures were approved by Shenzhen Third People's Hospital's Institutional Animal Care and Use Committee prior to the initiation of studies. All infectious works were performed under biosafety level 3 (BSL-3) conditions.

Infectious virus titration and viral load determination. The tissues from BALB/c mice including lungs, liver, spleen, kidneys, and ovaries were homogenized in 1 mL phosphate buffer solution (PBS) with 3 mm zirconium beads using a tissue homogenizer (Omni, Bead Ruptor 24 Elite). After two cycles of freeze-thaw, the lung tissue homogenates were centrifuged to remove tissue debris. Supernatants were transferred into fresh tubes.

Infectious viral titers were determined using focus forming assay. Briefly, the Vero E6 cells were seeded in 96 well plates and incubated overnight. Ten-fold serial dilutions of the MPXV were prepared in the maintenance medium. The Vero E6 cells were inoculated with serially diluted virus (100 µL/well). After 1 h of adsorption, the virus was removed and cells were washed once with PBS. Then 100 µL/well maintenance

medium was added. After 18 h incubation, the medium was aspirated and the cells were fixed with 4% paraformaldehyde solution for 30 min, permeabilized with Perm/Wash buffer (BD Biosciences) containing 0.1% Triton X-100, incubated with the HRP-conjugated anti-VACV polyclonal antibodies (Invitrogen) at room temperature for 2 hours. The reactions were developed with KPL TrueBlue Peroxidase substrates (Seracare Life Sciences). The number of virus foci was counted using an ELISpot reader (Cellular Technology Ltd), and the focus forming units (FFUs) per mL were calculated through the formula: average spots × virus dilution times×10 (FFU/mL).

MPXV viral loads (DNA copies) were determined using quantitative polymerase chain reaction (qPCR). Briefly, MPXV DNA in tissue homogenate supernatants was extracted using a nucleic acid extraction instrument (DaAn Gene, Smart 32) combined with Daan's extraction kit according to the manufacturer's instructions. The MPXV DNA copies were determined using SYBR Green Premix Pro Taq HS qPCR Kit (Accurate Biotech) with the following primers: forward primer 5'-TTT ATT CAA CAT GTA CTG TAC CCA C-3', reverse primer 5'- TTT CTT GCA TGG ATT TTC GTA TTT C -3'. MPXV B6R plasmid (Sangon Biotech) was serially diluted and performed to generate the standard curve.

ELISA. The 96-well plates (Conning, USA) were coated with 100 μL MPXV B6R or H3L recombinant proteins (Sino Biological, 2 μg/mL) overnight at 4 °C. Plates were washed with PBS and blocked with a blocking buffer (PBS containing 4% skim milk) at 37°C for 1 h. Mice serum samples were 100-fold diluted with the blocking buffer and added to the blocked plates, followed by incubation at 37 °C for 1 h. Plates were then washed three times with PBS-T (PBS containing 0.05% Tween-20) and incubated with goat anti-mouse IgG-HRP (1:20000, Abcam) at 37 °C for 40 min. Plates were washed with PBS-T three times, and HRP substrate TMB was added and developed at room temperature for 20 min. The reactions were stopped by 2M sulfuric acid. The absorbance at 450 nm was read using a Varioskan LUX multimode microplate reader (Thermo Fisher).

Focus reduction neutralization test (FRNT). To test the neutralization activity of serum from infected mice against MPXV, serum samples were collected from groups of mice 1/2/3 weeks post-infection and mock infection, respectively. Vero E6 cells were seeded at 1.5×10^4 cells/well into 96-well plates and used the following day. Heat-inactivated serum were 60-fold diluted with DMEM-2 medium containing 2% sterile guinea pig complement (Beijing Bersee Science and Technology Co.,Ltd). The serial dilutions of serum were mixed with an equal volume of diluted MPXV and then incubated at 37 °C for 1 h. The antibody-virus mixture was then added to the 96-well plate seeded with Vero E6 cells. The plates were incubated at 37 °C for about 16-18h. Then cells were fixed, permeabilized, stained, and developed, and the FFU was counted as aforementioned in the focus forming assay. Serum neutralization percentages were calculated using GraphPad Prism 8 (GraphPad Software Inc.).

Histopathological analysis. The middle lobe of the right lung of mice was fixed in 10% neutral buffered formalin for 24 hours, then processed with an automatic tissue hydroextractor (DAKEWE, HP300), embedded in paraffin, and sectioned at 3 μm for histological analysis. The Hematoxylin and Eosin (H&E) Staining was performed by a Tissue-Tek Prisma® Plus Automated Slide Stainer (Sakura Finetek). The photomicrographs of sections were taken using a slide scanning system (TEKSQRAY, SQS-600P). Stained slides were subsequently analyzed by a pathologist who was blinded to research groups.

Supplementary Figures

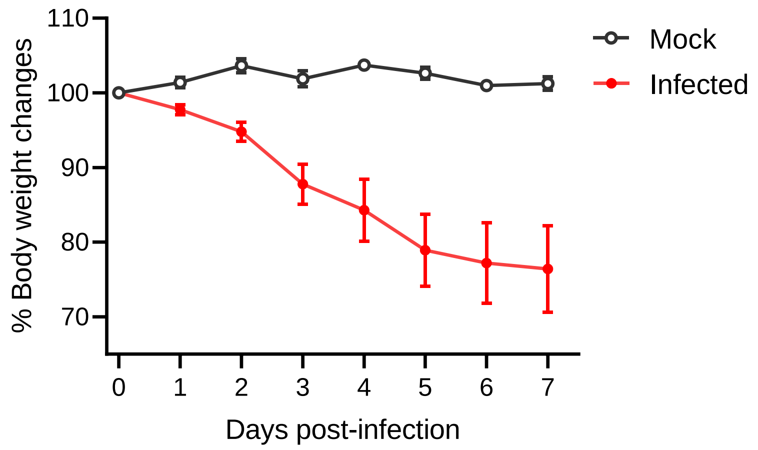

Supplementary Fig. S1. Groups of BALB/c mice (n = 5) were infected i.n. with MPXV strain SZTH42 (4×10^5 FFU in volume of 50 μL per mouse). Animals were monitored for weight loss for 7 days post-infection. Control mice were mock infected with 50 μL virus diluent.

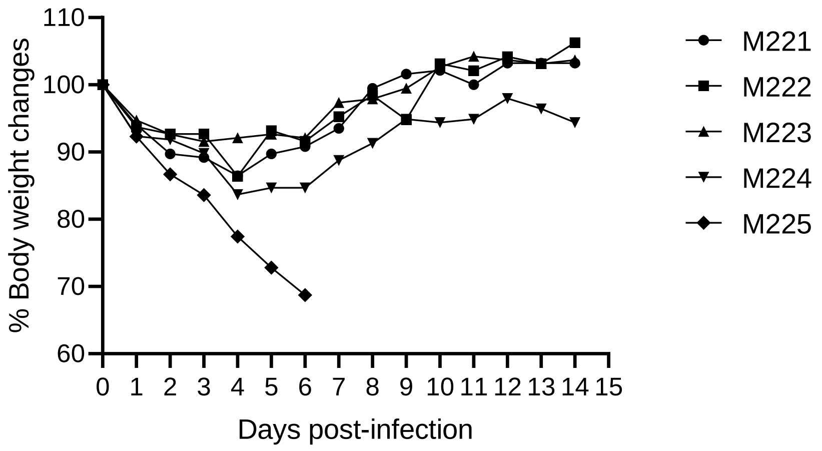

Supplementary Fig. S2. BALB/c mice (n = 5) were infected i.n. with MPXV SZTH42 (2×10^5 FFU in volume of 50 μL per mouse). Animals were monitored for weight loss for 14 days post-infection.

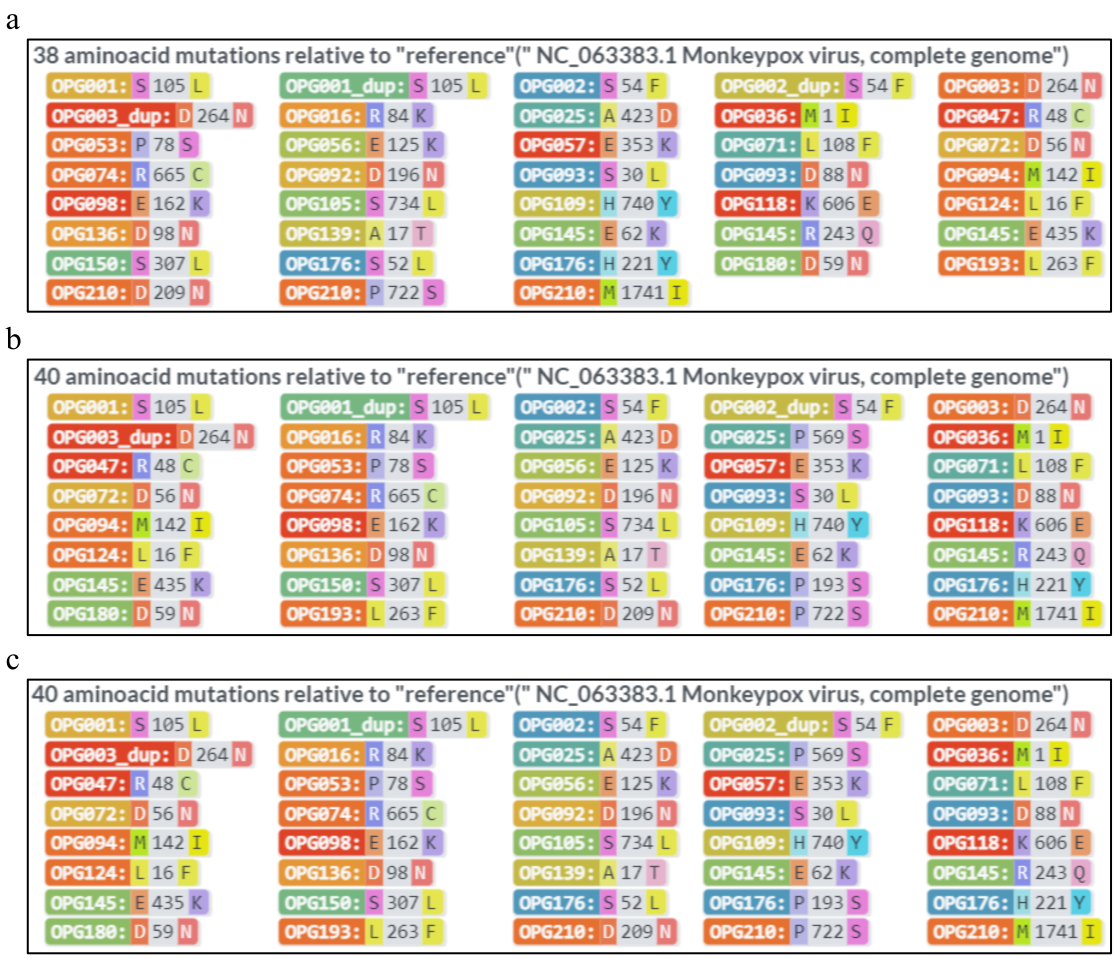

Supplementary Fig. S3. Alignment of MPXV isolates SZTH42 (a), SZTH41 (b), SZTH45 (c) with reference strain via Nextclade (https://clades.nextstrain.org/), genes and positions with amino acid mutations were shown.

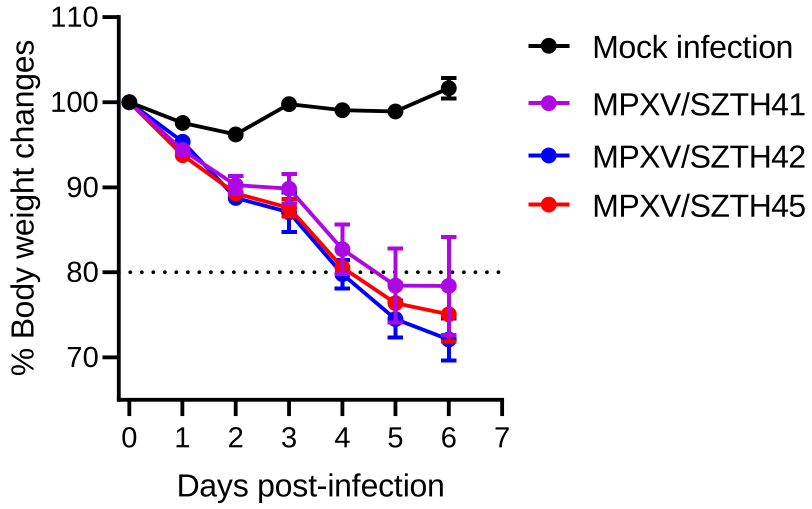

Supplementary Fig. S4. Groups of BALB/c mice (n = 5) were infected i.n. with MPXV isolates SZTH41, SZTH42 or SZTH45 (4×10^5 FFU per mouse). Animals were monitored for weight loss for 6 days post-infection. Control mice were mock infected with virus diluent.

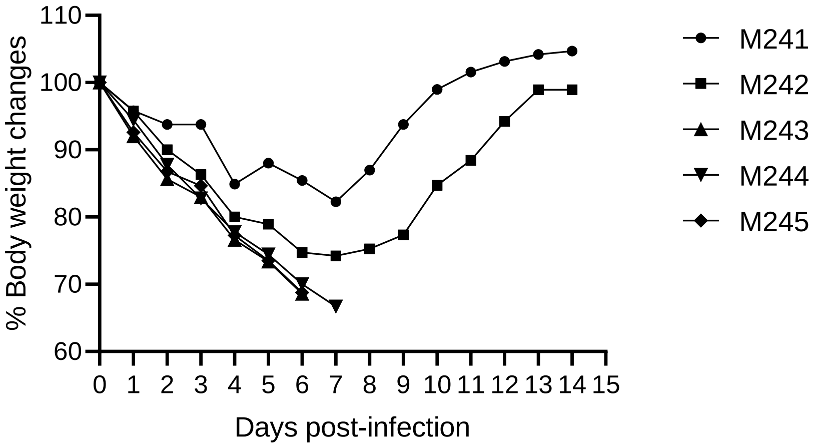

Supplementary Fig. S5. BALB/c mice (n = 5) were infected i.n. with MPXV SZTH42 (2×10^5 FFU in volume of 100 µL per mouse). Animals were monitored for weight loss for 14 days post-infection.

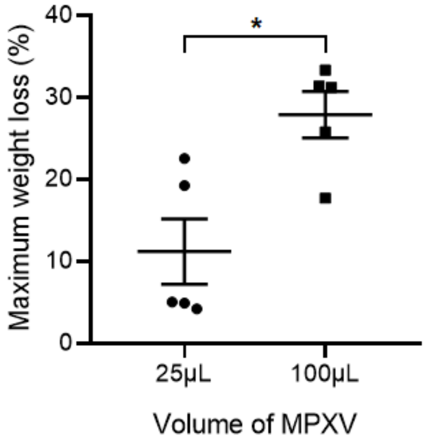

Supplementary Fig. S6. BALB/c mice (n = 5) were infected i.n. with MPXV SZTH42 (2×10^5 FFU in volume of 25 µL or 100 µL per mouse). Animals were monitored for weight loss for 14 days post-infection. The maximum weight loss was shown and statistically analyzed using Student's *t*-test. $^*P < 0.05$.

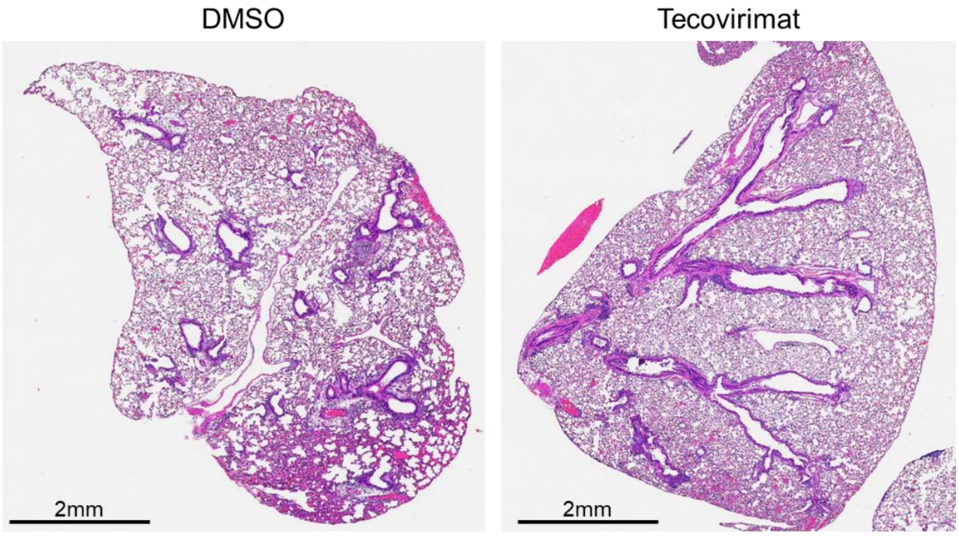

Supplementary Fig. S7. H&E staining of lung tissue sections at 4 dpi. Representative images are shown with low power.
